# Body composition assessment in a large cohort of Olympic athletes with different training loads: possible reference values for fat mass and fat-free mass domains

**DOI:** 10.1007/s00592-023-02203-y

**Published:** 2023-11-09

**Authors:** Luca Giovanelli, Giacomo Biganzoli, Antonio Spataro, Mara Malacarne, Giuseppina Bernardelli, Raffaella Spada, Massimo Pagani, Elia Biganzoli, Daniela Lucini

**Affiliations:** 1 BIOMETRA Department, University of Milan, Milan, Italy; 2 Medical Statistics Unit, Department of Biomedical and Clinical Sciences L. Sacco, “Luigi Sacco” University Hospital, University of Milan, Milan, Italy; 3 Sports Medicine Institute CONI, Rome, Italy; 4 DISCCO Department, University of Milan, Milan, Italy; 5 Exercise Medicine Unit, Istituto Auxologico Italiano, IRCCS, Milan, Italy

**Keywords:** Body composition, Olympic elite athletes, Fat mass, Fat-free mass, BOD POD, Cardiometabolic prevention and rehabilitation

## Abstract

**Aims:**

To assess body composition by means of BOD POD in the large cohort of Italian Olympic athletes of many sport disciplines (studied at the same time), and to provide possible reference values for body composition in elite athletes.

**Methods:**

1556 elite athletes, who took part in the selection procedure for the 2016 Rio Olympic Games for the National Italian Olympic Committee (CONI), were retrospectively studied. Body composition was determined using air plethysmography-based BOD POD.

**Results:**

We observed that Fat Mass (FM) and Fat-free Mass (FFM) should be considered as two mutually independent domains in elite athletes. By performing Principal Component Analysis, we defined two independent main domains (respectively, representing FM and FFM), which presented different trends according to gender and static or dynamic exercise load. Lastly, we reported possible reference values for FM index and FFM index, respectively, representing the largest contributors to FM domain and FFM domain, and calculated as FM or FFM (kg)/height (m^2^).

**Conclusions:**

Our findings might provide a basis to optimize the practical approach to body composition in athletes, highlighting the importance of considering indicators of fat mass and lean mass “simultaneously” and not specularly, according to different sport disciplines as well. Moreover, these data might contribute to standardize reference values for body composition in elite athletes, with a view to potentially helping to monitor and guide training regimens, prevent related detrimental practices and plan cardiometabolic prevention and rehabilitation programs.

## Introduction

In the setting of metabolic assessment, body composition represents a key parameter that should never be overlooked, being hierarchically superior to body mass index (BMI) in predicting disease risk [1]. However, focus on body composition rather than body weight still represents a crucial cultural challenge, especially in elite athletes [2, 3]. In particular, Olympic athletes represent a unique subset among top sport performers, since they follow tough training regimens in order to gain the first position on the world podium every fourth year. Along with multiple other factors (optimal physical training, psychological and autonomic regulation) [4], assessment of body composition may indeed help to monitor the quality of training regimens in order to ultimately optimize competitive performance, according to the specific sport discipline [5, 6].

Despite strong evidence for the importance of body composition in elite athletes, universally applicable reference values are to date still lacking. Several studies have been previously performed in this context [2, 5–11], featuring nonetheless a wide range of limitations, such as small sample size, enrollment of non-elite athletes, ethnic variability, issues with assessment methods (in terms of heterogeneity, reliability, invasiveness) and/or evaluation timing (in- or off-season training period).

Goal of the present study was to assess body composition by means of BOD POD in the large cohort of Italian Olympic athletes of many sport disciplines, studied at the same time, with a view to providing possible reference values for body composition in elite athletes.

## Methods

### Study population and methodology

This retrospective observational study involved data from 1556 elite athletes (99.2% of Caucasian ethnicity) (888 males and 668 females) (age 23.9 [19.6–28.6] and 22.6 [18.8–27.1], respectively) who took part in the selection procedure for the 2016 Rio Olympic Games for the National Italian Olympic Committee (CONI) [12]. All athletes were cleared for active sport participation. Thus, they were free from evidence of disease that could interfere with results. The assessment procedure took place at the Institute of Medicine and Sport Science (CONI, Rome), as part of the preparticipation screening, while data analysis was performed at the Exercise Medicine Unit, Istituto Auxologico Italiano, IRCCS, University of Milan.

**Fig. 1:**
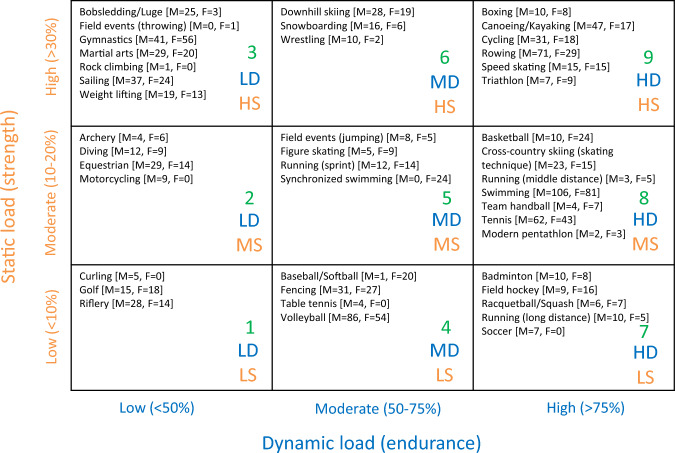
Classification of sports disciplines based on peak static and dynamic components achieved during competition (adapted from [13]). A progressive number (1–9) was given to each sport category. The increasing dynamic component is related to the estimated percentage of maximal oxygen uptake (VO_2max_) reached and results in an increasing cardiac output. The increasing static component is related to the estimated percentage of maximal voluntary contraction reached and results in an increasing blood pressure load. LD = low dynamic load; MD = moderate dynamic load; HD = high dynamic load; LS = low static load; MS = moderate static load; HS = high static load

The whole cohort was subdivided into nine groups according to Mitchell classification of sports disciplines [13], based on peak static and dynamic loads achieved during competition (Fig. 1).

Body composition was determined using air plethysmography-based BOD POD (Life Measurements Inc., Concord, CA, USA) according to the procedures recommended by the manufacturer [14, 15]. This noninvasive methodology was validated against other well recognized methods [16–18] in general adult and pediatric population as well as in athletes [2, 19–21]. Indeed, it was employed in many scientific papers in order to assess body composition as well as change in body composition after different training protocols both in athletes [19, 22–26] and non-athletes healthy subjects [27, 28]. The athletes were measured after at least 8 h of rest. When it was impossible to respect the 8 h of rest, the athlete was measured at least 2 h away from the ergometric test. Prior to each test, the BOD POD was calibrated according to the manufacturer’s instructions. While wearing minimal clothing (spandex shorts, swimsuit or tight-fitting underwear) and a swim cap, each subject was weighed on a calibrated digital scale to the nearest 20 g. The subject was then seated within the Air Displacement Plethysmography chamber for 2 measures of body volume, each lasting approximately 35–45 s. If the first 2 body volume values did not agree within 150 ml, a third body volume measure was taken. If 2 of the 3 measures were not consistent, the system was recalibrated and the test was repeated. Regarding the Thoracic Gas volume, we used predicted values, according to the standard predictor equation based on age, gender and height provided by the software [29]. The BOD POD was sealed and the participant breathed normally for 35–40 s while BV was measured. In stable resting conditions two measurements were obtained, and their average was utilized to provide body volume (BV, l) and body mass (BM, kg), as well as derive % fat mass (FM) and % fat-free mass (FFM) with specific equations (Siri, Brozek, Ortiz, Lohman, Schutte), according to ethnicity, gender and age.

In order to normalize the values of athletes’ FM and FFM for height, Fat Mass Index (FMi) and Fat-free Mass Index (FFMi) were considered [30] and calculated as follows:

- $${\text{FMi}} = {\text{FM}}\left( {{\text{kg}}} \right)/{\text{height}}\left( {{\text{m}}^{{2}} } \right)$$
- $${\text{FFMi}} = {\text{FFM}}\left( {{\text{kg}}} \right)/{\text{height}}\left( {{\text{m}}^{{2}} } \right)$$

The present study was performed in according to the principles of Declaration of Helsinki and Title 45, US Code of Federal Regulations, Part 46, Protection of Human Subjects, Revised November 13 2001, effective December 13 2001, and was cleared by local Institutional Science Committee (Istituto Medicina e Scienza dello Sport, Roma) and by Ethics Committee of University of Milan (report dated 23 September, 2019). The study protocol was guided by the STROBE statement [31].

### Statistical analysis

Continuous variables were presented as median (interquartile range). Categorical variables were presented as counts and percentages.

To visualize the shape of the distribution of the continuous variable analyzed, a nonparametric kernel density estimation method of the density function was applied, representing it by means of violin plots. The graphs were grouped by gender. This method allowed us to control whether specific patterns in the distributions of the variables were present. An examination of the summary statistics and boxplots would not highlight these patterns sufficiently.

As a preliminary step, to control for possible nonlinear non-monotonic associations between the variables studied, pairwise scatterplots and Pearson correlation coefficients were computed.

To jointly study the potential association between the anthropometric characteristics of the athletes, a principal component analysis (PCA) was performed.

This method allowed us to reduce the dimensionality of the dataset while preserving as much of the data variation as possible. This method consists of rotating the axes of the multivariate space of the original variables along orthogonal directions of maximal variance (principal components, PCs), and creating a new space defined by the PCs. Each of the PCs is characterized by a percentage of explained variance of the data. If a relevant amount of variance is explained by the first PCs, the projection of the variables as vectors on the subspace defined by the new axes can be useful in exploring correlation structures present in the data. If two variables are strongly correlated, they are projected close together (the correlation is positive) or conversely with maximum alignment (the correlation is negative). Otherwise, if they do not display correlation, they tend to be projected at an angle of 90 degrees. Moreover, if the subspace defined by the two principal components describes the variables well, these are projected toward a circle of radius unity, also known as the circle of correlation; otherwise, they tend to be projected close to the origin of the axes. In addition, it is possible to project individuals onto the new plane defined by the principal components to understand who shows similar multivariate patterns from those who show different ones.

By conducting this analysis, we tried to see how the nine Mitchell sports categories differentiate depending on the anthropometric variables studied. We analyzed the marginal trend of the first two principal components of each specific category by plotting the individual values of PC1 and PC2 versus the nine Mitchell sports categories and applying a nonparametric loess smoother. In line with the exploratory study objectives, no formal statistical testing was performed.

All the statistical analyses were conducted with R software (version 4.1.2).

#### Equity, diversity and inclusion statement

This study includes 888 males and 668 females athletes. They varied in gender, race and socioeconomic level. The authors are all Italian men and women, including 2 early career, 2 mid-career, 1 technician and 4 professors. The authors’ areas of expertise were exercise and sports medicine, endocrinology, internal medicine and behavioral medicine.

## Results

### Description of anthropometric variables

Table 1 reports the anthropometric variables of athletes stratified by gender. As expected, male athletes show greater values in all the variables except for FM, both in % and Kg, as compared to females.

**Table 1:** Anthropometric variables of athletes stratified by gender

*n*	Females	Males
	668	888
Age [yrs]	22.63 [18.84, 27.10]	23.95 [19.63, 28.61]
Height [cm]	169.00 [163.88, 174.00]	182.00 [176.00, 187.50]
BM [kg]	62.28 [56.53, 68.79]	77.84 [70.56, 86.52]
FM [kg]	12.09 [9.14, 15.17]	8.32 [5.68, 11.28]
FFM [kg]	49.86 [45.81, 54.63]	69.06 [62.90, 75.90]
FM [%]	19.25 [15.60, 22.72]	10.80 [7.80, 13.80]
FFM [%]	80.75 [77.27, 84.40]	89.20 [86.20, 92.20]
FM/FFM ratio	0.24 [0.18, 0.29]	0.12 [0.09, 0.16]
FMi [kg/m^2^]	4.20 [3.24, 5.25]	2.51 [1.75, 3.36]
FFMi [kg/m^2^]	17.60 [16.60, 18.52]	20.81 [19.56, 22.18]
BV [l]	59.22 [53.55, 65.30]	72.56 [65.47, 80.64]
BD [kg/l]	1.05 [1.05, 1.06]	1.07 [1.07, 1.08]
BSA [m^2^]	17,081 [16217, 18211]	19,939 [18690, 21211]

Continuous variables are reported as median and interquartile range, whereas categorical variables as frequencies and percentage

*BM*—body mass, *FM*—fat mass, *FFM*—fat-free mass, *FMi*—fat mass index, *FFMi*—fat-free mass index, *BV*—body volume, *BD*—body density, *BSA*—body surface area

Table 2 summarizes the anthropometric variables of athletes stratified by Mitchell sport category (Fig. 1).

**Table 2:** Anthropometric variables of athletes stratified by Mitchell sport category

2a. Females	1	2	3	4	5	6	7	8	9
*n*	32	29	117	101	52	27	36	178	96
Age [yrs]	25.36 [18.93, 32.41]	26.98 [20.35, 31.05]	20.35 [17.18, 24.20]	23.29 [20.23, 28.04]	23.52 [20.88, 27.80]	25.00 [22.03, 31.03]	26.06 [20.28, 29.07]	21.78 [16.50, 25.60]	23.26 [21.74, 26.58]
Height [cm]	165.30 [162.00, 169.25]	164.00 [160.00, 168.00]	164.00 [155.00, 171.00]	176.00 [170.00, 182.30]	169.50 [164.00, 172.00]	168.00 [164.85, 170.00]	164.90 [161.25, 170.00]	170.00 [166.00, 175.38]	168.85 [165.00, 173.00]
BM [kg]	62.10 [56.49, 68.86]	59.94 [55.55, 64.14]	56.62 [50.46, 62.52]	69.15 [64.36, 74.24]	57.16 [53.22, 60.92]	63.35 [60.79, 70.68]	58.88 [52.36, 63.10]	64.11 [58.92, 70.26]	63.55 [60.13, 68.75]
FM [kg]	16.29 [12.29, 20.21]	12.65 [10.21, 16.72]	9.97 [5.78, 13.76]	13.37 [10.62, 16.57]	8.18 [5.86, 10.98]	13.78 [9.62, 15.63]	11.27 [7.85, 13.49]	12.17 [10.00, 15.43]	12.42 [10.62, 15.20]
FFM [kg]	45.05 [42.30, 48.64]	46.69 [43.10, 49.57]	45.44 [41.58, 51.12]	54.74 [50.08, 60.76]	48.56 [45.20, 51.58]	51.05 [48.21, 54.61]	47.05 [44.72, 50.11]	51.48 [47.94, 55.63]	51.02 [47.96, 54.01]
FM [%]	26.50 [22.58, 30.35]	21.40 [18.50, 26.20]	17.40 [12.80, 22.10]	20.10 [15.80, 22.70]	14.55 [10.30, 19.00]	20.40 [15.75, 22.55]	18.65 [15.33, 21.42]	19.20 [16.33, 22.70]	19.65 [17.32, 22.52]
FFM [%]	73.50 [69.65, 77.43]	78.60 [73.80, 81.50]	82.60 [77.90, 87.20]	79.90 [77.30, 84.20]	85.45 [81.00, 89.70]	79.60 [77.45, 84.25]	81.35 [78.57, 84.67]	80.80 [77.30, 83.68]	80.35 [77.47, 82.67]
FM/FFM	0.36 [0.29, 0.44]	0.27 [0.23, 0.36]	0.21 [0.15, 0.28]	0.25 [0.19, 0.29]	0.17 [0.12, 0.23]	0.26 [0.19, 0.29]	0.23 [0.18, 0.27]	0.24 [0.20, 0.29]	0.24 [0.21, 0.29]
FMi [kg/m^2^]	6.04 [4.46, 7.06]	4.58 [3.72, 6.12]	3.69 [2.26, 4.95]	4.51 [3.34, 5.20]	2.98 [2.03, 3.91]	4.94 [3.59, 5.32]	4.16 [3.10, 4.72]	4.20 [3.52, 5.15]	4.29 [3.76, 5.27]
FFMi [kg/m^2^]	16.72 [15.51, 17.40]	17.17 [15.90, 18.57]	17.06 [15.88, 18.34]	17.83 [17.02, 18.71]	17.28 [16.37, 18.22]	18.40 [17.59, 19.22]	17.27 [16.55, 17.94]	17.74 [16.98, 18.55]	18.02 [17.23, 18.59]
BV [l]	59.87 [53.84, 66.61]	56.54 [52.64, 61.47]	53.60 [47.40, 59.34]	65.22 [60.82, 70.64]	53.71 [50.31, 57.65]	60.16 [57.13, 67.82]	56.07 [49.19, 60.24]	60.91 [55.81, 66.82]	60.18 [56.92, 65.45]
BD [kg/l]	1.03 [1.03, 1.04]	1.05 [1.04, 1.05]	1.06 [1.05, 1.07]	1.05 [1.05, 1.06]	1.07 [1.05, 1.08]	1.05 [1.05, 1.06]	1.05 [1.05, 1.06]	1.05 [1.05, 1.06]	1.05 [1.05, 1.06]
BSA [m^2^]	16,609 [16403, 17822]	16,458 [15857, 17319]	16,170 [14955, 17297]	18,475 [17443, 19488]	16,619 [16031, 17052]	17,158 [16647, 18067]	16,479 [15417, 17277]	17,413 [16592, 18447]	17,172 [16613, 18251]

2b. Males	1	2	3	4	5	6	7	8	9
*n*	48	54	152	122	25	54	42	210	181
Age [yrs]	28.11 [23.92, 33.35]	26.26 [20.01, 38.17]	22.76 [19.38, 28.59]	21.91 [18.38, 26.98]	26.37 [23.88, 28.71]	27.20 [23.69, 30.05]	27.27 [20.60, 30.60]	21.02 [16.73, 26.48]	24.72 [21.10, 28.24]
Height [cm]	176.90 [173.88, 183.25]	175.50 [172.57, 180.00]	176.50 [170.50, 183.00]	192.00 [183.00, 200.00]	183.00 [178.00, 185.00]	178.00 [175.00, 183.75]	179.25 [176.00, 184.88]	184.00 [179.00, 189.00]	183.00 [178.20, 189.00]
BM [kg]	79.41 [73.16, 89.00]	73.63 [64.91, 79.23]	74.25 [67.64, 82.82]	84.99 [77.38, 90.71]	78.13 [75.66, 80.94]	81.16 [73.20, 85.64]	71.05 [66.01, 74.81]	77.60 [69.53, 85.82]	79.36 [72.47, 89.22]
FM [kg]	14.85 [10.56, 21.63]	10.18 [6.26, 14.13]	7.56 [4.87, 10.24]	9.34 [6.56, 11.31]	5.71 [5.05, 6.50]	7.95 [5.33, 12.62]	7.15 [4.69, 8.70]	8.01 [5.70, 10.78]	8.51 [6.00, 10.91]
FFM [kg]	63.98 [58.74, 68.51]	61.85 [56.92, 66.87]	66.31 [61.15, 73.33]	73.91 [68.58, 80.81]	71.19 [69.47, 75.14]	70.61 [66.00, 75.26]	63.12 [60.49, 68.08]	69.10 [62.93, 76.01]	70.43 [64.72, 79.54]
FM [%]	20.50 [14.10, 25.90]	14.75 [9.50, 18.55]	10.20 [7.27, 13.10]	10.95 [8.30, 13.57]	7.30 [6.50, 8.50]	10.00 [7.70, 14.67]	9.50 [6.93, 12.30]	10.60 [7.73, 13.28]	10.70 [8.00, 13.20]
FFM [%]	79.50 [74.10, 85.90]	85.25 [81.45, 90.50]	89.80 [86.90, 92.73]	89.05 [86.43, 91.70]	92.70 [91.50, 93.50]	90.00 [85.32, 92.30]	90.50 [87.70, 93.07]	89.40 [86.73, 92.27]	89.30 [86.80, 92.00]
FM/FFM	0.26 [0.16, 0.35]	0.17 [0.10, 0.23]	0.11 [0.08, 0.15]	0.12 [0.09, 0.16]	0.08 [0.07, 0.09]	0.11 [0.08, 0.17]	0.10 [0.07, 0.14]	0.12 [0.08, 0.15]	0.12 [0.09, 0.15]
FMi [kg/m^2^]	4.78 [3.45, 6.99]	3.30 [2.12, 4.64]	2.39 [1.65, 3.26]	2.51 [1.78, 3.06]	1.69 [1.54, 1.94]	2.56 [1.77, 3.81]	2.14 [1.43, 2.67]	2.43 [1.77, 3.12]	2.55 [1.84, 3.23]
FFMi [kg/m^2^]	20.03 [18.94, 20.83]	19.87 [19.10, 21.06]	21.32 [20.28, 22.97]	20.77 [19.33, 21.89]	21.39 [20.32, 21.79]	22.28 [20.35, 22.99]	19.58 [18.67, 21.10]	20.45 [19.40, 21.87]	21.26 [20.13, 22.68]
BV [l]	75.49 [68.49, 85.43]	69.08 [60.42, 74.48]	69.67 [62.33, 77.56]	79.38 [72.05, 84.42]	71.81 [69.90, 74.80]	75.64 [67.84, 80.34]	66.42 [60.86, 69.20]	72.07 [64.45, 80.13]	74.06 [67.40, 82.94]
BD [kg/l]	1.05 [1.04, 1.07]	1.06 [1.06, 1.08]	1.08 [1.07, 1.08]	1.07 [1.07, 1.08]	1.08 [1.08, 1.09]	1.08 [1.07, 1.08]	1.08 [1.07, 1.08]	1.07 [1.07, 1.08]	1.07 [1.07, 1.08]
BSA [m^2^]	19,863 [18824, 20914]	18,907 [17864, 19799]	19,021 [17855, 20626]	21,298 [20169, 22501]	19,998 [19713, 20413]	19,973 [18653, 20987]	18,847 [18299, 19703]	20,026 [18723, 21217]	20,166 [19079, 21582]

Continuous variables are reported as median and interquartile range, whereas categorical variables as frequencies and percentage

*BM*—body mass, *FM*—fat mass, *FFM*—fat-free mass, *FMi*—fat mass index, *FFMi*—fat-free mass index, *BV*—body volume, *BD*—body density, *BSA*—body surface area

### Principal component analysis (PCA)

Considering the large number of parameters derived from body composition assessment, as well as the fact that some of them were mutually correlated, and that simple pairwise associations did not consider the joint relationships with the other variables, a thorough investigation of the association structure among the parameters was performed by exploiting principal component analysis (PCA). This was done to look at the amount of information (indicated by the proportion of variance explained by each principal component in %) that was carried by relevant experimental parameters. Starting from the entire set of data, PCA produced a smaller number of uncorrelated components. These components may suggest hypothesis about the nature of the modeling data structure, and attribute to groups of parameters a physiological meaning.

The principal component analyses were conducted for males and females separately.

The structure of correlation in the dataset considered the following variables: FMi and FFMi (derived from FM and FFM Kg, and height), FFM%, FM%, BD, BSA, BV, FM/FFM ratio, and age.

The cumulative proportion of variance explained by the first two principal components was 90%, so only these were thoroughly investigated.

**Fig. 2:**
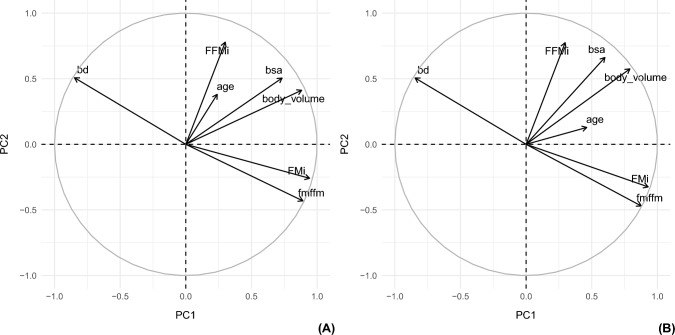
Considering the relevant amount of variance explained by the first dimensions, the projection of the variables as vectors on the subspace defined by the new axes can be useful in exploring their correlation structure. From the graph both for Females (**A**) and Males (**B**) FMi and FFMi do not display correlation, as they are projected at an angle of 90 degrees. Body density (bd) is inversely correlated with FMi but is not correlated with FFMi. As expected, BSA and Body Volume are highly correlated, as FMi and FM-FFM ratio (fmffm). Age is not well defined by PC1 and PC2 as its vector is closer to the origin

While PC1 was characterized by FMi, FFM%, FM%, FM/FFM ratio, and BD variables, PC2 was characterized by FFMi, BSA, and BV variables. Figure 2 depicts the correlation structure of the data in females (A) and males (B) by projecting the variables in the space defined by the two dimensions. It is noteworthy that the variables FMi and FFMi form an angle of 90° degrees and so they seem uncorrelated. Moreover, the largest contributors for the characterization of PC1 and PC2 are FMi (22%) and FFMi (35%), respectively.

Since the nature of PCA requires that the dimensions extracted are uncorrelated with each other, and since FMi and FFMi are the largest contributors to PC1 and PC2, respectively, we could state that FMi and FFMi were mutually independent.

**Fig. 3:**
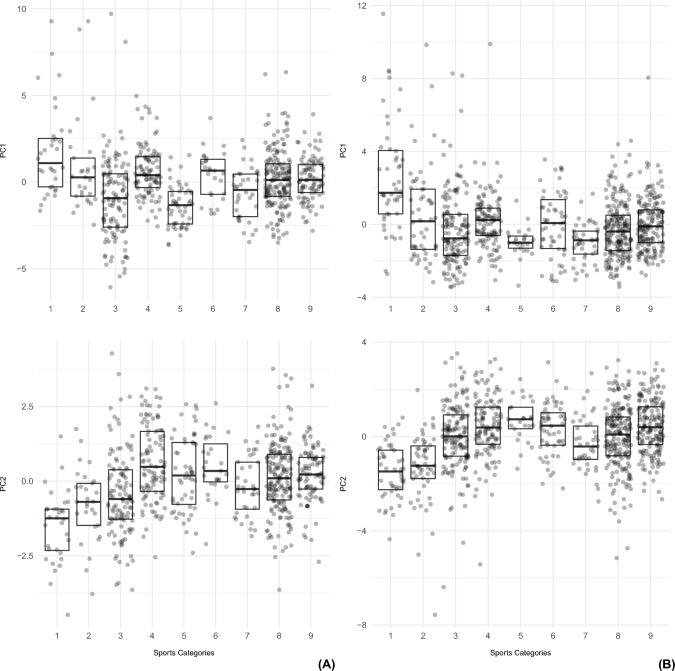
Trend of PC1 and PC2 values across the nine sport categories (according to Mitchell classification) in females (**A**) and males (**B**)

Figure 3 reports in two different scatterplots, both for females (A) and males (B), the trend of PC1 and PC2 values (including FMi and FFMi as the most representative variables, respectively) depending on the nine sport categories.

Regarding females (Fig. 3A), in disciplines with a low dynamic load (LD, cat 1, 2, 3, see Fig. 1), PC1 values decreased while PC2 values increased, as the static load increased. Instead, in categories with a moderate (MD, cat 4, 5, 6 see Fig. 1) and high dynamic load (HD, 7, 8, 9 see Fig. 1), the two PCs both increased, with the exception of category 5, which exhibited a significant decrease in both PC1 and PC2.

In males (Fig. 3B), the average values of PC1 decreased drastically in disciplines with a low dynamic load (LD, cat 1, 2, 3, see Fig. 1) from category 1 to 3 (as the static load increased), and then settled at low values up to category 9 (as the dynamic load increased). Notably, categories 6 and 9 were characterized by a slight increase in PC1 values, while the lowest average values were found in categories 5 and 7. PC2 steadily increased from category 1 to 3, reached maximum values in categories 4 to 6 (characterized by a moderate dynamic load), then it went down in category 7, and rose again as the static load increased.

Based on the above results—considering that FMi and FFMi are the largest contributors to PC1 and PC2, respectively, that they are mutually independent, and can be easily calculated in clinical practice—we tried to derive reference values for male and female elite athletes’ body composition according to sport discipline, with a view to monitoring and guiding training regimes of current and future athletes.

Table 3 summarizes average FMi and FFMi values in the nine sports categories. We observed that low static load (LS) disciplines are characterized by a decrease in FMi values with the increase in dynamic component (from Cat 1 to Cat 4, and Cat 7) both in males and females, while FFMi values remain substantially the same. Moderate static load (MS) disciplines are characterized by a different trend of FMi values, being lower in presence of moderate dynamic load (MD) (Cat 5) as compared to both low (LD) and high dynamic load (HD) (Cat 2 and Cat 8, respectively) both in males and females. On the contrary, FFMi values are similar across the three dynamic load levels in females and slightly higher in moderate dynamic load (MD) in males.

**Table 3:**
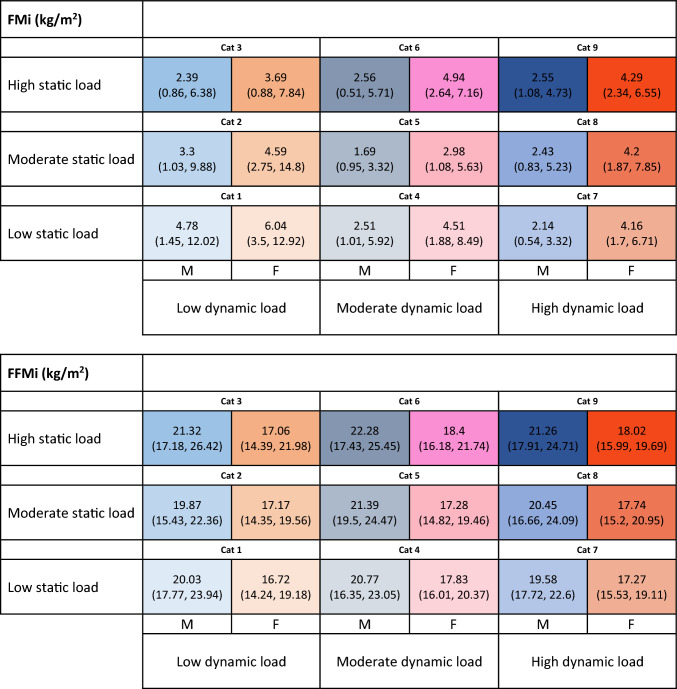
Summary of average FMi and FFMi values in nine sports categories (according to Mitchell classification)

Data are reported as median (2.5 percentile, 97.5 percentile)

*FMi* fat mass index, *FFMi* fat-free mass index, *M* males, *F* females

High static load (HS) disciplines are characterized by similar values of FMi across the three dynamic load levels (Cat 3, Cat 6, and Cat 9) in males, and by higher FMi values in moderate dynamic load (MD) disciplines (Cat 6) in females. Indeed, FFMi values in males are similar in low (LD) and high dynamic load (HD) categories and slightly higher in moderate dynamic load (MD) disciplines, while in females they are similar in moderate (MD) and high dynamic load (HD) categories and slightly lower in low dynamic load (LD) disciplines.

## Discussion

In this paper, we assessed body composition by means of BOD POD in a large cohort of Olympic athletes of many sport disciplines studied at the same time, and we observed that FM and FFM should be considered as two mutually independent domains in elite athletes. By performing Principal Component Analysis, we defined two independent main domains (respectively, representing FM and FFM), which show different trends according to gender and static or dynamic exercise load. Lastly, we reported possible reference values for FMi and FFMi, representing the largest contributors to FM domain and FFM domain, respectively.

Body composition plays a crucial role in elite athletes, and improvements in body composition have been found to enhance both cardiorespiratory fitness and strength [6]. Notably, body composition is also related to the risk of eating disorders and unhealthy weight management practices, which are oft due to widespread unawareness and misperception, leading to extremes in underweight or—to a lesser extent—overweight, especially among weight sensitive sports performers [3]. In this respect, it is worth mentioning the female athlete triad (isolate or combined presence of amenorrhea, impaired bone mass, and eating disorders), with each component existing on a spectrum from optimal health to disease. This condition, arising from low energy availability, mainly affects girls practicing sports that require leanness [32, 33].

However, universally applicable reference values for body composition in elite athletes are to date still lacking. Several studies have been previously performed in this context [2, 5–11], featuring nonetheless a wide range of limitations, such as small sample size, enrollment of non-elite athletes, ethnic variability, issues with assessment methods (in terms of heterogeneity, reliability, invasiveness) and/or evaluation timing (in- or off-season training period). With particular regards to assessment of body composition, all the available techniques present some limitations. For instance, reliability of skinfold thickness measurement depends on the technician’s skill and the caliper’s brand; bioelectrical impedance analysis (BIA) is population-specific, it is influenced by multiple variables, and may underestimate/overestimate FFM; dual-energy X-ray absorptiometry (DXA) is highly reliable but expensive and associated with a small amount of radiation [16]. In this study, BOD POD was employed, which can be considered as the best noninvasive technology, allowing to obtain reasonably accurate measurement of essential elements of body composition (FM and FFM, both expressed in absolute physical units, kilograms, and nominal % value) [19, 34]. Based on air plethysmography, it is indeed simple, friendly, and somewhat inexpensive [2, 16]. Although disease states can affect its accuracy, this is not a concern for elite athletes, being free from evidence of disturbances. Notably, this methodology has been also validated in athletes [20, 21, 35], and well-employed to assess body composition as well as body mass changes after different training protocols both in athletes and general population [19, 22–28]. Further, it was even used as reference methodology to assess the validity and reliability of other new proposed techniques in athletes [36–39].

We observed, by employing advanced statistics, that two simple Indexes (FMi and FFMi) [30, 40–43] were the largest contributors to PC1 and PC2, respectively, representing FM domain and FFM domain. According to such findings, FMi and FFMi, easily calculated from noninvasive body composition assessment, might have a considerable potential in clinical practice.

Thus, we leveraged these two Indexes to analyze the trend of FM and FFM across the nine Mitchell sports categories. In particular, FMi looks higher in athletes who perform disciplines with low dynamic component (LD), such as riflery and archery, while FFMi was lower in the same athletes as compared to other disciplines. This mirrored trend of FM and FFM is not present in some of the other sport categories, where FM and FFM seem to be two separate domains with mutually independent trends in both genders. In particular, athletes from category 7 (e.g., long-distance running) present not only low FM but also relatively low absolute values of FFM; conversely, athletes from category 9 (e.g., canoeing) present not only high FFM but also a considerable amount of FM. This observation may be of particular interest from a clinical point of view in monitoring athlete’s body composition. While in general population FFM is usually derived as percentage difference from FM, this cannot be applied to many categories of elite athletes. In this setting, FM and FFM need in fact to be considered separately. On the contrary, in the usual clinical practice FM and FFM are considered as two specular variables, with the consequent risk of missing some important information that is indeed necessary to help athletes reach/maintain their optimal body composition. This approach could be particularly relevant in identifying and managing athletes—especially those who perform weight sensitive sports—with risk for eating disorders and unhealthy weight management practices, which are oft due to widespread unawareness and misperception, leading to extremes in underweight or—to a lesser extent—overweight [3]. The possibility to have reference values both for FMi and FFMi from a relatively large population of elite athletes of many sport disciplines may further give a contribution to this goal.

### Clinical implications

Our findings might provide a basis to optimize the practical approach to body composition in athletes, highlighting the importance of considering indicators of fat mass and lean mass “simultaneously” and not specularly, according to different sport disciplines as well. Moreover, these data might contribute to standardize reference values for body composition in elite athletes according to sports disciplines. Assessment of body composition in athletes could potentially help to monitor and guide training regimens of current and future athletes as well as prevent related detrimental practices (identification of at-risk athletes). This latter aspect also could be prominent among non-elite athletes playing sports that tend to emphasize low body weight, and it could help to plan cardiometabolic prevention and rehabilitation programs.

### Limitations

Firstly, we have neither data regarding all sport disciplines nor a sufficient number of athletes in some of them. Consequently, we must consider nine main sport categories as indicated by international classification [13]. Indeed, we present data on the largest population of elite athletes in Italy, assessed using the same methodology, by the same researchers and at the same training epoch. Secondly, the percentage of female and male athletes in some sport disciplines is different (for instance, we have no male athletes performing synchronized swimming).

## Data Availability

Data are available upon reasonable request. We do not upload the data, considering the paucity of subjects in some sport disciplines (elite athletes well known by the community), which may not guarantee privacy.
